# Supplementary material for: Decision rules for identifying combination therapies in open‐entry, randomized controlled platform trials
Source: Pharm Stat. 2022 Jan 31;21(3):671–90. doi: 10.1002/pst.2194 (PMC9304586; doi:10.1002/pst.2194)
Supplement: Supplementary file 1 — Appendix S1: Supporting information [file PST-21-671-s001.pdf]

Supplemental Material for: Decision rules for identifying combination therapies in open-entry, randomized controlled platform trials

Elias Laurin Meyer[1], Peter Mesenbrink[2], Cornelia Dunger-Baldauf[3], Ekkehard Glimm[3,4], Yuhan Li[2], and Franz König[1,*]

on behalf of EU-PEARL (EU Patient-cEntric clinicAl tRial pLatforms) Consortium

[1] *Center for Medical Statistics, Informatics, and Intelligent Systems, Medical University of Vienna, Austria*
[2] *Novartis Pharmaceuticals Corporation, One Health Plaza, East Hanover, NJ, USA*
[3] *Novartis Pharma AG, Basel, Switzerland*
[4] *Institute of Biometry and Medical Informatics, University of Magdeburg, Germany*
[*] *Correspondence: franz.koenig@meduniwien.ac.at; Tel.: +43-1-40400-74800*

1 Further Treatment Efficacy Scenarios

In the main text, we showed nearly exclusively selected results of one set of assumptions regarding the treatment effect of the monotherapies and the combination treatment (setting 1). In general, we only investigated treatment effect assumptions based on risk-ratios, whereby we randomly and separately draw the risk-ratio for each of the monotherapies with respect to the SoC treatment. For the combination treatment, we randomly draw from a range of interaction effects, which could result in additive, synergistic or antagonistic effects of a specified magnitude. Some scenarios might be more realistic for a given drug development program than others, however we felt that the broad range of scenarios will allow to investigate the impact and interaction of the various simulation parameters and assumptions on the operating characteristics. Let π_x denote the probability of a patient on therapy x to have a successful treatment outcome (binary), i.e. the response-rate, and T_x denote a discrete random variable.

In detail, every time a new cohort enters the platform, we firstly fix the SoC response-rate:

$$\pi_{SoC} \in [0, 1]$$

Then we assign the treatment effect in terms of risk-ratios for the backbone monotherapy (monotherapy A), which is the same across all cohorts:

$$\pi_{MonoA} = \pi_{SoC} * \gamma_{MonoA}, \gamma_{MonoA} \sim T_{MonoA}$$

Then we randomly draw the treatment effect in terms of risk-ratios for the add-on monotherapy (monotherapy B):

$$\pi_{MonoB} = \pi_{SoC} * \gamma_{MonoB}, \gamma_{MonoB} \sim T_{MonoB}$$

Finally, after knowing the treatment effects of both monotherapies, we randomly drew an interaction effect for the combination treatment:

$$\pi_{Combo} = \pi_{SoC} * (\gamma_{MonoA} * \gamma_{MonoB}) * \gamma_{Combo}, \gamma_{Combo} \sim T_{Combo}$$

Depending on the scenario, the distribution functions can have all the probability mass on one value, i.e. the assignment of treatment effects and risk-ratios is not necessarily random. Please further note that while the treatment effects were specified in terms of risks and risk-ratios, the Bayesian decision rules were specified in terms of response rates. Two settings characterize global null hypotheses, six settings characterize an

efficacious backbone monotherapy with varying degrees of add-on mono and combination therapy efficacy, two settings characterize an efficacious backbone with varying degrees of random add-on mono and combination therapy efficacy, two settings characterize either the global null hypothesis or efficacious mono and combination therapies, but with an underlying time-trend, and two settings were run as sensitivity analyses with increased standard-of-case response rates. The different treatment efficacy settings are summarized in table 1.

1.1 Impact of add-on monotherapy and combination therapy efficacy

For settings 2-7 we assumed the backbone monotherapy to be efficacious (response rate of 20% throughout, compared to SoC with a response rate of 10%). The add-on monotherapy and the combination therapy treatment effects are varied independently of each other (add-on monotherapy either 10% or 20% response rate and combo therapy response rate varying from 20% to 40%). Figure 1 shows the results. For cases where the add-on monotherapy response rate is 0.1 and/or the combination therapy response rate is 0.2, only type 1 error related error rates are shown, as - according to our definition - there exist no correct positive and false negative decisions under these circumstances. Similarly, for the rest of the scenarios, only power related operating characteristics are shown. It could be argued whether in some cases, in which the combination therapy is highly efficacious, even if one of the components is not superior to SoC, investigators would want to declare the cohort successful and not consider this a type 1 error. In settings 3-6, the PCT1ER and the FWER increase with increasing final cohort sample size. This is most likely due to the combined decision rule, where apparently the probability to declare the combination (correctly) superior to the mono therapies increases faster than the probability to declare the add-on monotherapy (correctly) not superior to SoC. This is due to our definition of the error rates, whereby any incorrect decision in the individual comparisons leads to an overall incorrect decision, even though 3/4 decisions might be correct. In settings 7 and 8, we observe an increase in per-cohort power and disjunctive power when the cohort sample size increases. Both are maximized when pooling all data and minimized when sharing no data.

1.2 Global null scenario

We investigated two global null scenarios, (i) with the response rates of all arms equal to 0.10 (setting 1) and (ii) with the response rates of all arms are equal to 0.20 (setting 2). Results are presented in figure 2. As in these settings no true positive or false negative decision can be made, only PCT1ER and FWER in terms of error rates are presented. The PCT1ER generally slightly decreases with increasing number of cohorts in the platform trial when we use data sharing (as would be expected since we are using Bayesian decision rules and share more data). In most cases the PCT1ER decreases so slowly with increasing maximum number of cohorts that as a result the FWER increases. When we share no data we would expect no impact of the number of cohorts on the PCT1ER. We believe the slight fluctuations we observe might be well within the expected simulation error as we are dealing with rare events (e.g. a FWER of 0.001 translates to at least one false positive decision in 10 out of 10000 simulated platforms). In terms of data sharing, we mostly observe the lowest error rates when using the dynamic borrowing approach. Generally, as the decision rules being used are quite conservative, we observe negligible type 1 error rates both on the platform and cohort level for the global null scenario. However, we did not want to use more lenient decision rules, as under partial null scenarios (see figure 1), we see a substantial FWER and PCT1ER inflation.

1.3 Impact of time trend

Finally, we looked at two situations where a time trend in response rates exists that affects all treatment arms (e.g. improvement of SoC). Firstly, we consider a global null hypothesis where all the response rates are 10% + (c-1)*3, whereby c denotes the cohort number in the trial, meaning within the first cohort all treatment arms have a response rate of 10%, in the second cohort all treatments have a response rate of 13% etc. Secondly, we assume a true alternative hypothesis where the SoC response rates are 10% + (c-1)*3, the monotherapy response rates are 20% + (c-1)*3 and the combination therapy response rates are 40% + (c-1)*3. These two situations correspond to treatment efficacy settings 13 and 14. Results are presented in figure 3. For setting 11, only type 1 error related operating characteristics are shown (as there are no true positive decisions), while for setting 12 only power related operating characteristics are shown (as there are no false positive

Table 1: Overview of different treatment effect assumptions. The priors T_{MonoA}, T_{MonoB} and T_{Comb} for γ_{MonoA}, γ_{MonoB} and γ_{Comb} are all pointwise with a support of 1,2 or 3 different points (each with probability "p") and result in effective response rates π_{SoC}, π_{MonoA}, π_{MonoB} and π_{Comb}. Only results of setting 1 are shown in the main text, the rest in the supplements.

Setting	π_{SoC}	π_{MonoA} (γ_{MonoA})	π_{MonoB} (γ_{MonoB})	π_{Combo} (γ_{Combo})	Description
1	0.10	0.20 (2)	0.10 (1) with p 0.5<br>0.20 (2) with p 0.5	0.20 (1) if $\gamma_{MonoB} = 1$<br>0.40 (1) if $\gamma_{MonoB} = 2$	backbone monotherapy superior to SoC, add-on monotherapy has 50:50 chance to be superior to SoC; in case add-on monotherapy not superior to SoC, combination therapy as effective as backbone monotherapy, otherwise combination therapy significantly better than monotherapies
2	0.10	0.20 (2)	0.10 (1)	0.20 (1)	backbone monotherapy superior to SoC, but add-on monotherapy not superior to SoC and combination therapy not better than backbone monotherapy
3	0.10	0.20 (2)	0.10 (1)	0.30 (1.5)	backbone monotherapy superior to SoC and combination therapy superior to backbone monotherapy, but add-on monotherapy not superior to SoC
4	0.10	0.20 (2)	0.10 (1)	0.40 (2)	backbone monotherapy superior to SoC and combination therapy superior to backbone monotherapy (increased combination treatment effect compared to setting 4), but add-on monotherapy not superior to SoC
5	0.10	0.20 (2)	0.20 (2)	0.20 (0.5)	both monotherapies are superior to SoC, but combination therapy is not better than monotherapies
6	0.10	0.20 (2)	0.20 (2)	0.30 (0.75)	both monotherapies are superior to SoC and combination therapy is better than monotherapies
7	0.10	0.20 (2)	0.20 (2)	0.40 (1)	both monotherapies are superior to SoC and combination therapy is superior to monotherapies (increased combination treatment effect compared to setting 7)
8	0.10	0.10 (1)	0.10 (1)	0.10 (1)	global null hypothesis
9	0.20	0.20 (1)	0.20 (1)	0.20 (1)	global null hypothesis with higher response rates
10	0.10	0.20 (2)	0.10 (1) with p 0.5<br>0.20 (2) with p 0.5	0.20*γ_{MonoB}*0.5 (0.5) with p $\frac{1}{3}$<br>0.20*γ_{MonoB}*1 (1) with p $\frac{1}{3}$<br>0.20*γ_{MonoB}*1.5 (1.5) with p $\frac{1}{3}$	backbone monotherapy superior to SoC, add-on monotherapy has 50:50 chance to be superior to SoC; combination therapy interaction effect can either be antagonistic/non-existent, additive or synergistic (with equal probabilities)
11	0.10 + 0.03*(c-1)	0.10 + 0.03*(c-1) (1)	0.10 + 0.03*(c-1) (1)	0.10 + 0.03*(c-1) (1)	time-trend null scenario; every new cohort (first cohort $c = 1$, second cohort $c = 2$, ...) will have SoC response rate that is by 3%-points higher than that of the previous cohort
12	0.10 + 0.03*(c-1)	0.20 + 0.03*(c-1) (2)	0.20 + 0.03*(c-1) (2)	0.40 + 0.03*(c-1) (1)	time-trend scenario, whereby monotherapies superior to SoC and combination therapy superior to monotherapies; every new cohort (first cohort $c = 1$, second cohort $c = 2$, ...) will have SoC response rate that is by 3%-points higher than that of the previous cohort
13	0.20	0.30 (1.5)	0.30 (1.5)	0.40 ($\frac{8}{9}$)	analogous to setting 7, but SoC response rate is 20%
14	0.20	0.30 (1.5)	0.30 (1.5)	0.50 ($\frac{10}{9}$)	analogous to setting 8, but SoC response rate is 20%

decisions). With few exceptions, we observe consistently higher PCT1ER and FWER when sharing more data. This shows that when borrowing from other sources (e.g. from other cohorts, both concurrent and non-concurrent) the type 1 error is negatively affected. We also observe that disjunctive power is relatively lower when sharing all data compared to sharing less data (compare with figure 5 in the main text).

1.4 Impact of decision rules at interim

In this section we look at the impact of 1) not allowing for early stopping at interim for futility and 2) using a short-term surrogate endpoint for interim decision making. For ease of result interpretation, we used the final endpoint for interim decision making by default in the main text. However, there are many scenarios in which this will not be efficient or feasible (e.g. when the final endpoint is a long-term endpoint and by the time 50% of the patients have reached this endpoint, 100% of the planned sample size of patients have already been enrolled and started treatment). Therefore, we also investigate scenarios in which a short-term surrogate endpoint is used for interim decision making. We define the quality of the short-term endpoint in terms of its sensitivity and specificity (e.g. 90%) to be predicted by the final endpoint (i.e., if y_{ij} are the interim ($j = 0$) and final ($j = 1$) responses of patient i, then $P(y_{i0} = 1|y_{i1} = 1) = P(y_{i0} = 0|y_{i1} = 0) = 0.9$). Conceptually it would make more sense to specify the sensitivity and specificity of the interim outcome in predicting the final outcome (i.e. $P(y_{i1} = 1|y_{i0} = 1)$ and $P(y_{i1} = 0|y_{i0} = 0)$), however from a probabilistic perspective this would put constraints on the final response rate with respect to the chosen sensitivity and specificity (otherwise the probability of observing an interim outcome could be smaller than 0), making e.g. a final response rate of 0.1, together with a sensitivity of 90 % and specificity of 85 % impossible. Since we believe the main quantity of interest is the final response rate, and we wanted to avoid having to double check for every combination of final response rate and sensitivity/specificity whether it is feasible, we chose to specify the sensitivity and specificity of the final outcome in predicting the interim outcome. To be specific, when the final response rate is x% and sensitivity and specificity of the final outcome in predicting the interim outcome are set to se% and sp% respectively, the joint probability distribution for the interim and final event is as follows: $P(y_{i1} = 1, y_{i0} = 1) = se * x$, $P(y_{i1} = 1, y_{i0} = 0) = (1 - se) * x$, $P(y_{i1} = 0, y_{i0} = 1) = (1 - sp) * (1 - x)$, $P(y_{i1} = 0, y_{i0} = 0) = sp * (1 - x)$.

The impact of the quality of the short-term endpoint and disallowing early stoping for efficacy is shown in figure 4 and uses treatment efficacy scenario 10. For setting 10, the backbone monotherapy (response rate 20%) is superior to SoC (response rate 10%) and the add-on monotherapy efficacy is random, with 50% probability to be as efficacious as the backbone monotherapy (response rate 20%) and 50% probability to be not efficacious (response rate 10%). Building on top of the monotherapies, the combination interaction effect is random (see table 1). As expected, when we disallow early stopping for futility, the quality of the short-term endpoint has no impact on the power. When we allow early stopping for futility, we see that the quality of the short-term endpoint has a major impact on both the per-cohort and per-platform power. As an example: When we use no data sharing, the PCP is roughly 25% when the sensitivity and specificity as described above are 0.65, in contrast to roughly 75% when the sensitivity and specificity are 1 (i.e. the final endpoint is used for interim decision making). The results in figure 4 also show a clear difference between Disj_Power and Disj_Power_BA. Please remember from table 1 in the main text that error rates without the ending "BA" are defined as proportions of platform trials where the hypothesis of interest was true for at least one cohort, e.g. when computing the FWER, we divide the number of platform trials with at least one false positive decision by the number of platform trials where at least one cohort was in truth not efficacious. In the "BA" error rates, we disregard whether or not the hypothesis of interest was true for at least one cohort and always divide by the total number of platform trials simulated. This can be interpreted as taking the prior distribution on the effectiveness of the treatments into account. No "BA" error rates are always increased compared to "BA" error rates, as we would expect since the denominator is smaller. In some cases, since in setting 10 it is more likely for a new cohort to be not efficacious according to our definitions, Disj_Power_BA is even lower than PCP.

Funding

EU-PEARL (EU Patient-cEntric clinicAl tRial pLatforms) project has received funding from the Innovative Medicines Initiative (IMI) 2 Joint Undertaking (JU) under grant agreement No 853966. This Joint Undertaking receives support from the European Union's Horizon 2020 research and innovation programme and EFPIA and Children's Tumor Foundation, Global Alliance for TB Drug Development non-profit organisation, Springworks Therapeutics Inc. This publication reflects the authors' views. Neither IMI nor the European Union, EFPIA, or any Associated Partners are responsible for any use that may be made of the information contained herein. The PhD research of Elias Laurin Meyer was funded until 11/2020 by Novartis through the University and not at an individual level.

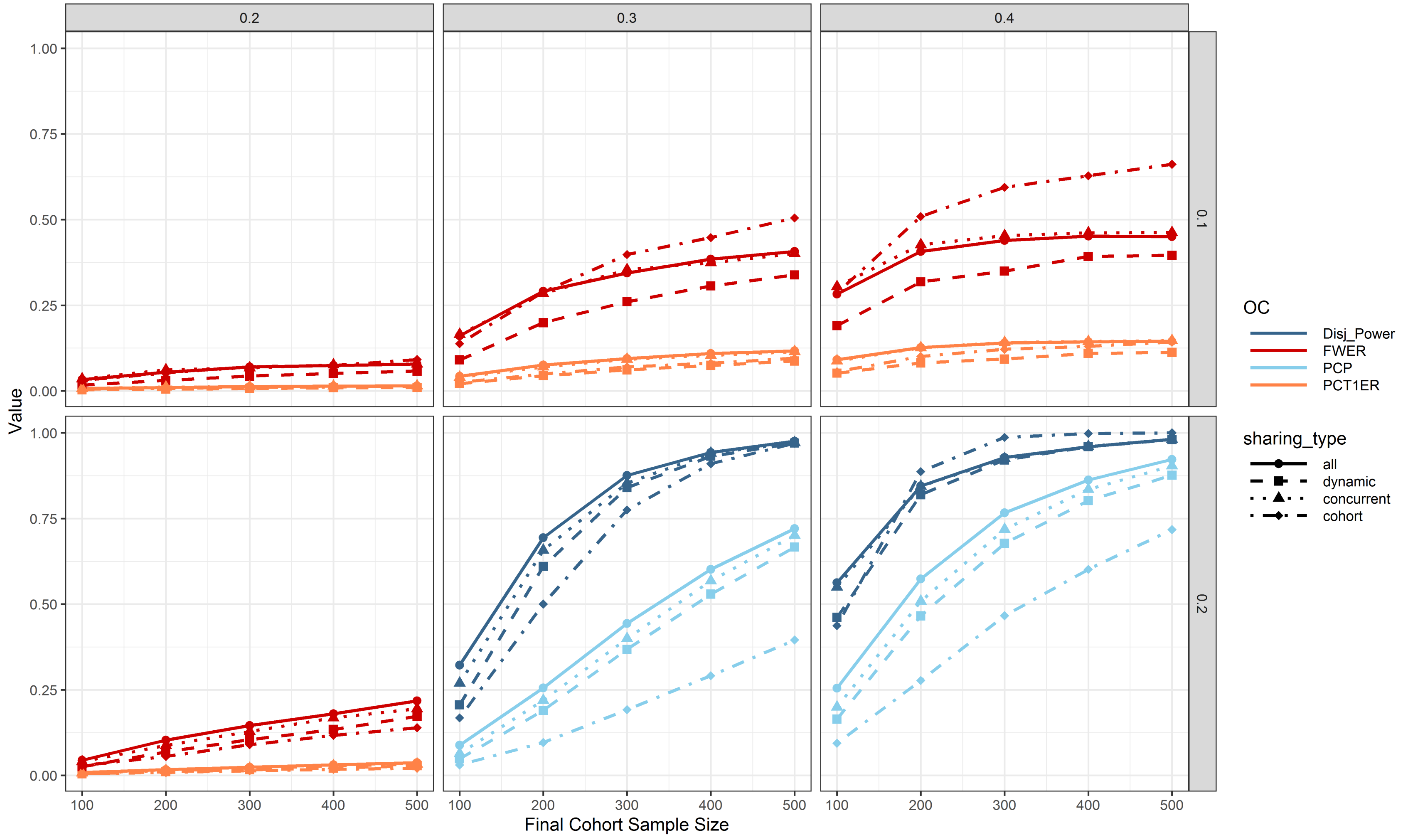

Figure 1: Operating characteristics (OC) of treatment efficacy settings 2-7 with respect to planned final cohort sample size (x-axis), type of data sharing (linetype and point shape), response rate of combination arm (columns) and response rate of add-on monotherapy (rows) (e.g. the top left panel corresponds to treatment efficacy setting 3 and the bottom right panel corresponds to treatment efficacy setting 8). For cases where the add-on monotherapy response rate is 0.1 and/or the combination therapy response rate is 0.2, only type 1 error related error rates are shown. Similarly, for the rest of the scenarios, only power related operating characteristics are shown.

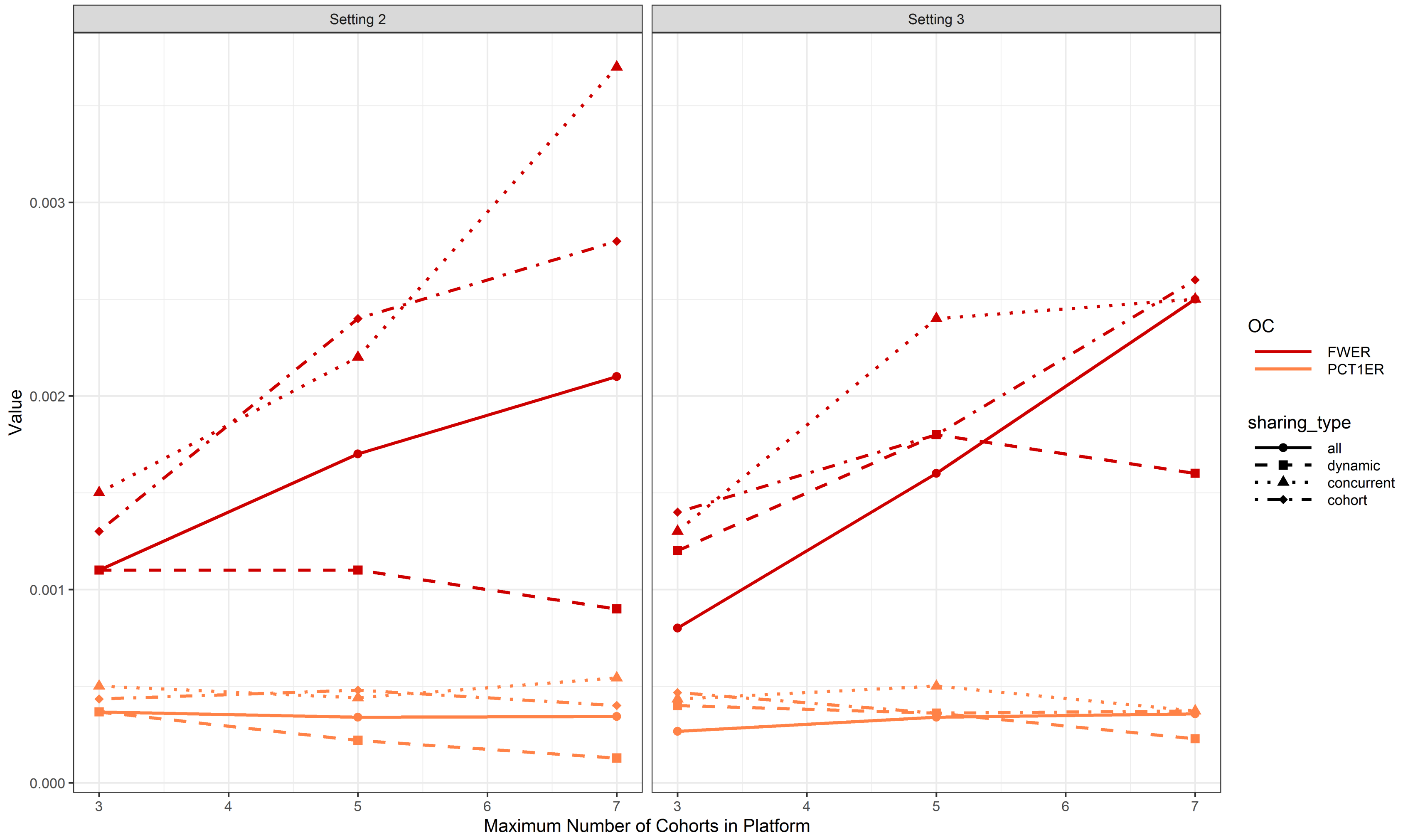

Figure 2: Operating characteristics (OC) for treatment efficacy settings 1 and 2 with respect to maximum number of cohorts (x-axis), setting (columns) and type of data sharing (linetype and point shape). The PCT1ER generally slightly decreases with increasing number of cohorts in the platform trial when we use data sharing, however so slowly that the FWER still increases. Generally, as the decision rules being used are quite conservative, we observe negligible type 1 error rates both on the platform and cohort level for the global null scenario.

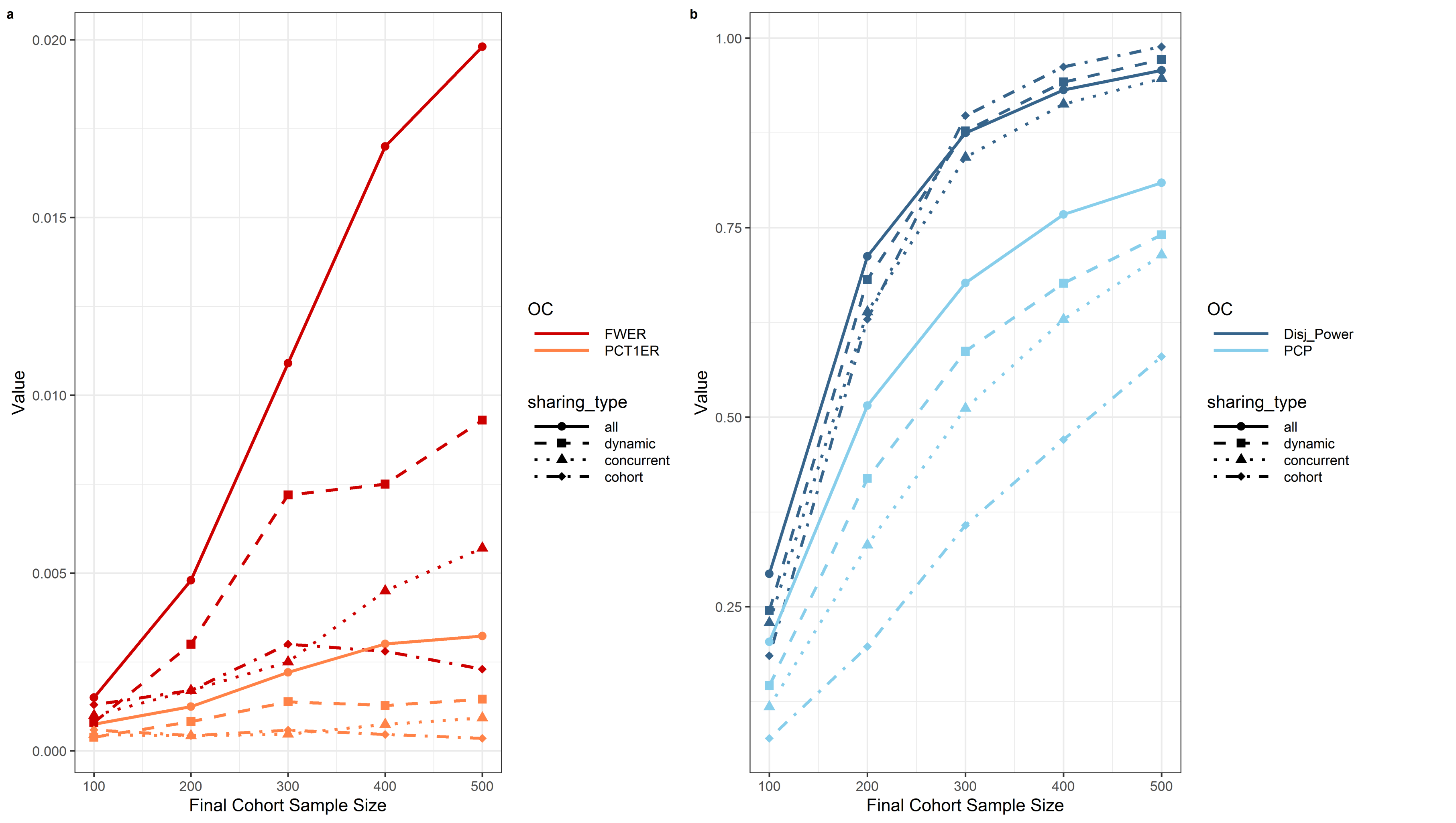

Figure 3: Operating Characteristics (OC) of time trend settings 11 (a) and 12 (b). Linetypes and point shapes represent different data sharing types. For setting 13, only type 1 error related operating characteristics are shown (as there are no true positive decisions), while for setting 14 only power related operating characteristics are shown (as there are no false positive decisions). In most cases, type 1 error rates are greatly increased when sharing more non-concurrent data. PCP does not profit as much from sharing data as when no time trend is present (compare with figure 1).

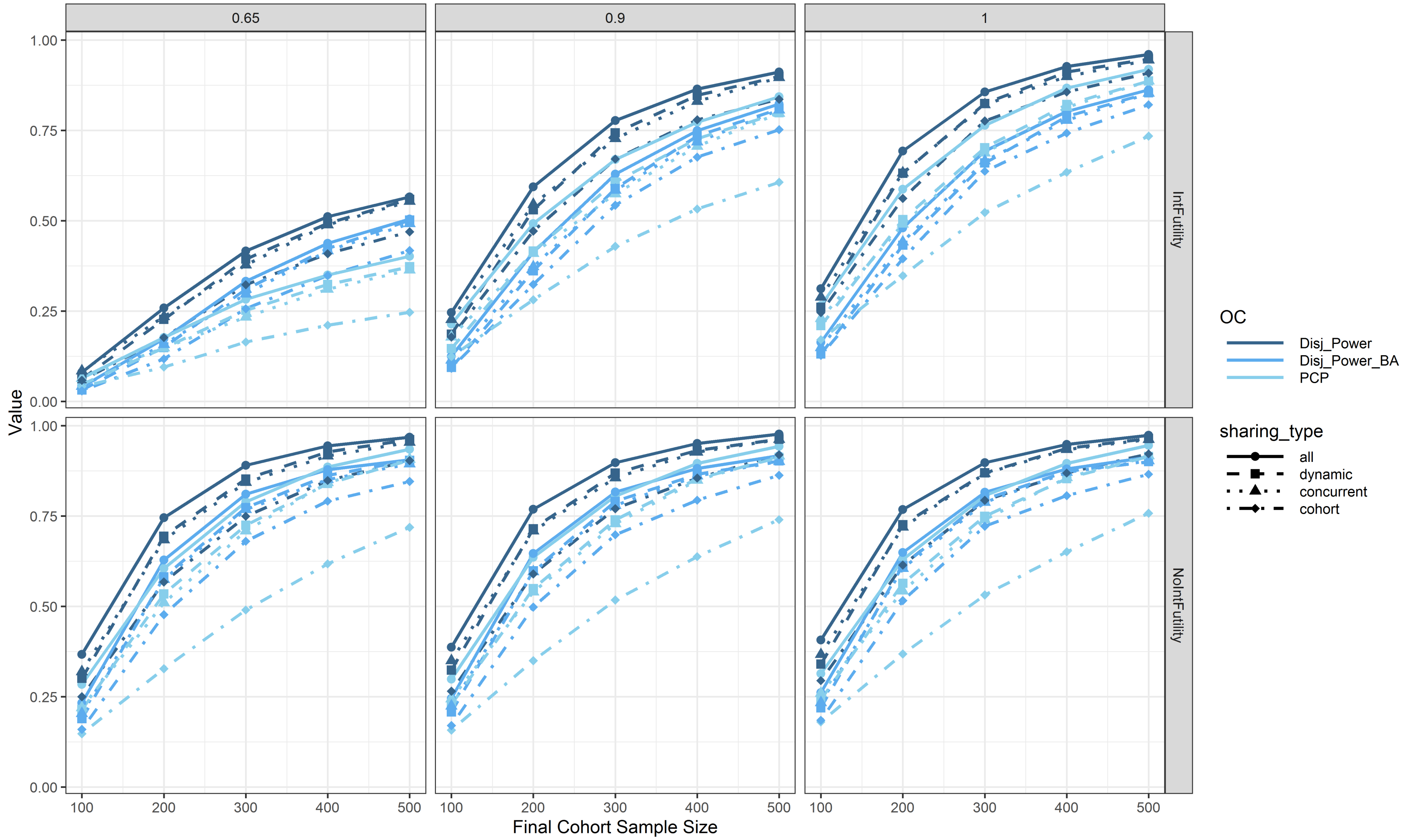

Figure 4: Operating characteristics (OC) of treatment efficacy setting 10 with respect to planned final sample size (x-axis), type of data sharing (linetype and point shape), quality of the short-term endpoint used at interim (columns) and whether or not we allow stopping at interim for early futility (rows). While in case we do not stop at interim for futility, the quality of the short-term endpoint used has no impact on the power, in case of stopping at interim for futility, a substantial power loss can be observed if the quality of the short-term endpoint used is low. The results also show a clear difference between Disj_Power and Disj_Power_BA.
